# Overcoming Nanoscale Inhomogeneities in Thin-Film Perovskites via Exceptional Post-annealing Grain Growth for Enhanced Photodetection

**DOI:** 10.1021/acs.nanolett.1c03839

**Published:** 2022-01-21

**Authors:** Tian Du, Filipe Richheimer, Kyle Frohna, Nicola Gasparini, Lokeshwari Mohan, Ganghong Min, Weidong Xu, Thomas J. Macdonald, Haozhen Yuan, Sinclair R. Ratnasingham, Saif Haque, Fernando A. Castro, James R. Durrant, Samuel D. Stranks, Sebastian Wood, Martyn A. McLachlan, Joe Briscoe

**Affiliations:** † School of Engineering and Materials Science and Materials Research Institute, Queen Mary University of London, London E1 4NS, United Kingdom; ‡ National Physical Laboratory, Hampton Road, Teddington TW11 0LW, United Kingdom; § Cavendish Laboratory, JJ Thomson Avenue, Cambridge CB3 0HE, United Kingdom; ∥ Department of Chemistry and Centre for Processable Electronics, Imperial College, London W12 0BZ, United Kingdom; ⊥ Department of Materials and Centre for Processable Electronics, Imperial College, London W12 0BZ, United Kingdom; ○ SPECIFIC IKC, College of Engineering, Swansea University, Swansea SA2 7AX, United Kingdom; ∇ Department of Chemical Engineering & Biotechnology, University of Cambridge, Philippa Fawcett Drive, Cambridge CB3 0AS, United Kingdom

**Keywords:** Thin-film perovskites, nanoscale inhomogeneities, grain growth, photoresponse, photodetection

## Abstract

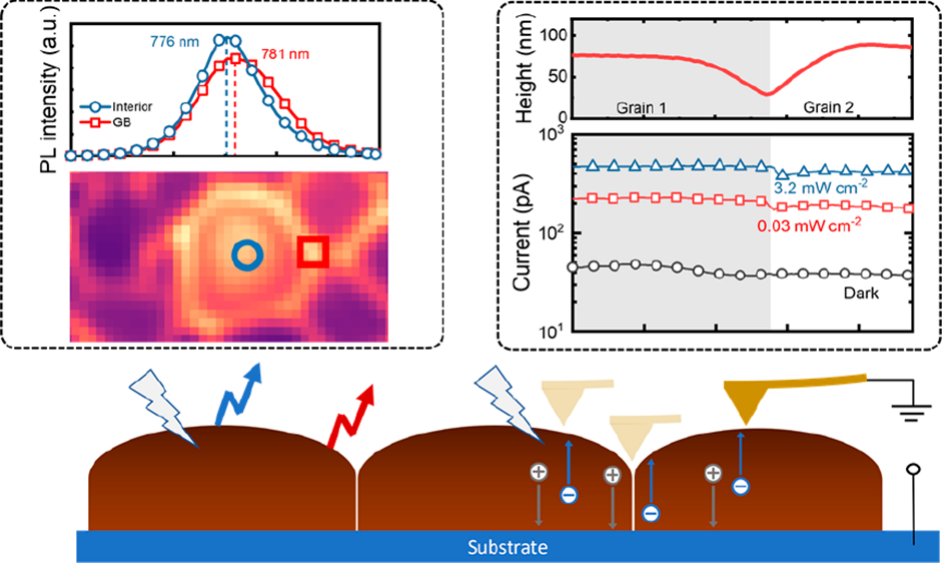

Antisolvent-assisted spin coating has been widely used for fabricating metal halide perovskite films with smooth and compact morphology. However, localized nanoscale inhomogeneities exist in these films owing to rapid crystallization, undermining their overall optoelectronic performance. Here, we show that by relaxing the requirement for film smoothness, outstanding film quality can be obtained simply through a post-annealing grain growth process without passivation agents. The morphological changes, driven by a vaporized methylammonium chloride (MACl)–dimethylformamide (DMF) solution, lead to comprehensive defect elimination. Our nanoscale characterization visualizes the local defective clusters in the as-deposited film and their elimination following treatment, which couples with the observation of emissive grain boundaries and excellent inter- and intragrain optoelectronic uniformity in the polycrystalline film. Overcoming these performance-limiting inhomogeneities results in the enhancement of the photoresponse to low-light (<0.1 mW cm^–2^) illumination by up to 40-fold, yielding high-performance photodiodes with superior low-light detection.

Solution-processed metal halide perovskites (MHPs) are inherently polycrystalline, consisting of crystallographic^1^ and electronic defects.^2^ Although high-performance solar cells can be achieved from these solution-processed films,^3^ as the trap states in MHPs are reported to be less detrimental under solar irradiance,^4^ the impact of trap-mediated recombination is non-negligible at low carrier densities.^5,6^ Under weak illumination, for example, trap-mediated recombination may completely switch off charge transfer to contact layers.^4,7^ Hence, the control of crystallographic defects and thus electronic trap states in thin-film MHPs is of great importance to devices working in the dark or under low-light conditions, such as transistors,^8^ photodetectors,^9^ or indoor solar cells.^10^

Since first being introduced,^11^ the “antisolvent washing” for spin coating of perovskite films has become the dominant technique used for high-performing devices. The overriding factor driving the uptake of this method is the need for smooth, compact, and planar perovskite films for subsequent deposition of charge transport layers by solution-processed routes. However, the rapid perovskite crystallization induced by antisolvent washing and subsequent thermal annealing gives rise to relatively small grains (∼150–200 nm in diameter). These small grains typically demonstrate a strongly heterogeneous shape owing to limited space for postcrystallization growth. As such, local inhomogeneity exists in these films in terms of material composition,^12^ crystallographic defects,^13^ trap density,^14^ and lattice strains^15^ with the local inhomogeneity being observed not only intergrain^16,17^ but also intragrain.^18,19^ In addition, the prevalent grain boundaries (GBs) in polycrystalline films are heterogeneous to the grain interior in nature,^20,21^ and they typically host a variety of crystallographic defects.^22^ Therefore, overcoming the local inhomogeneities, particularly at the GBs, is a critical route toward improvement of the overall quality of polycrystalline films.

Post-deposition crystal growth is widely used to improve the grain sizes of thin-film polycrystalline semiconductors above the film thickness.^23^ For MHP films, this has been realized through elevated temperature,^24^ high-energy laser pulses,^25^ or solution-assisted Ostwald ripening.^26,27^ However, the poor stability of perovskites^22^ poses a fundamental limit to the time and temperature windows for post-deposition treatments. Herein, we report a solid-state secondary grain growth method enabling a nearly 10-fold increase of lateral grain size at a typical thermal annealing temperature for MAPbI_3_ perovskites (100 °C) within a short period of time (5 min). The treatment drives a comprehensive morphological transition toward minimization of surface energy, enabling not only the novel photophysical observation of emissive GBs but also exceptional nanoscale photoconductive uniformity and superior low-light photoresponse, leading to high-performance photodiodes.

## Results and Discussion

**Figure 1:**
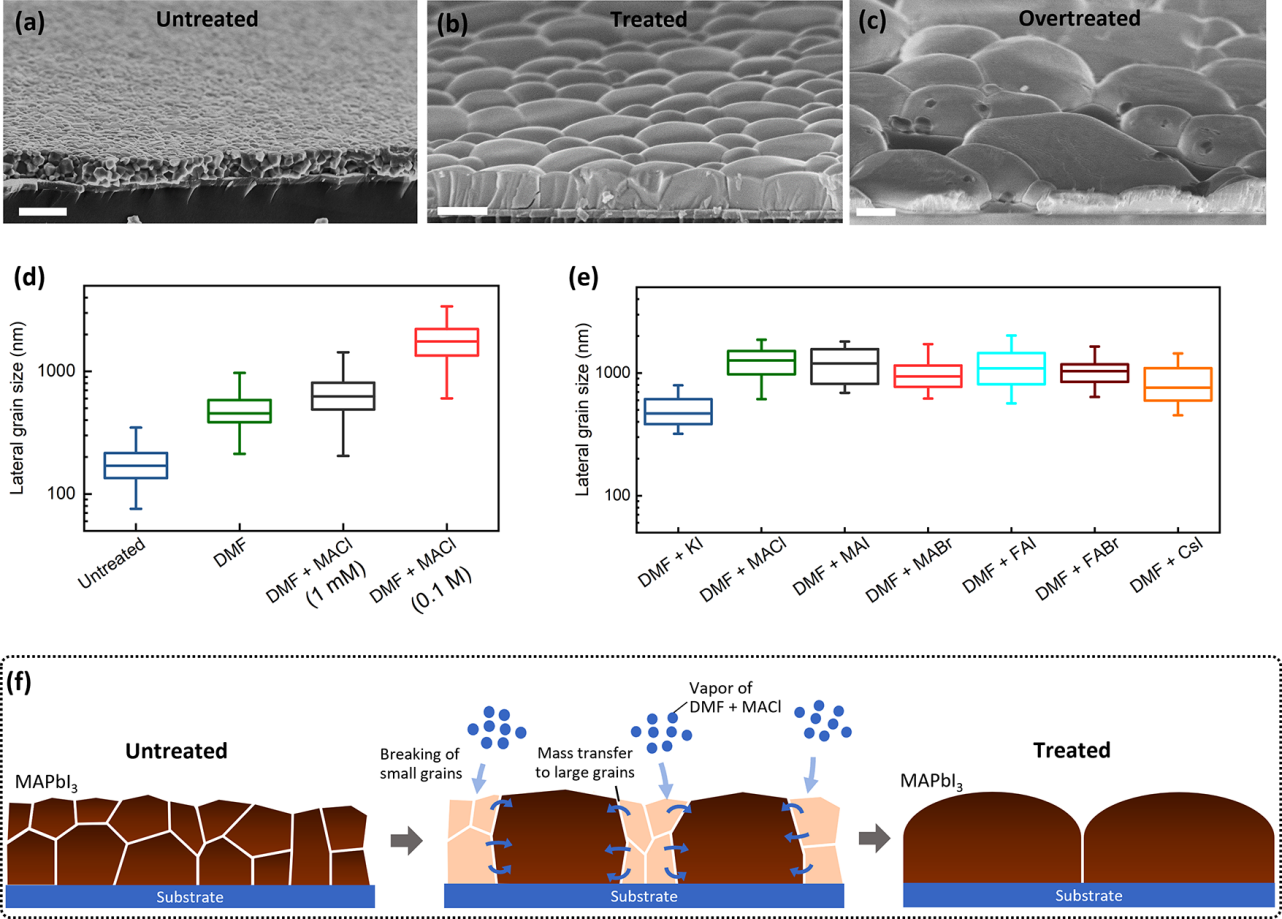
Grain growth of MAPbI_3_ films induced by treatment with DMF–MACl aerosol. (a–c) Tilt-angle cross-section SEM images of untreated MAPbI_3_ film after antisolvent-assisted spin coating (a), treated MAPbI_3_ film for 5 min, (b) and overtreated MAPbI_3_ film for 10 min (c). Scale bars in all images are 500 nm. (d) Impact of MACl concentration in DMF on the statistical data of lateral grain size. (e) Impact of different halide compound additives in DMF (all with concentrations of 0.1 M) on the statistical data of lateral grain size. (f) Schematic drawing of MAPbI_3_ film morphological transition during MACl treatment and the process of aerosol ingression to the MAPbI_3_ films; growth of larger MAPbI_3_ grains at the expense of surrounding smaller grains.

The perovskite (MAPbI_3_) films are deposited through an established “antisolvent washing” method followed by thermal annealing.^28^ Post-deposition treatment of the MAPbI_3_ films was carried out in an aerosol-assisted chemical vapor deposition setup^29^ by passing through aerosolized dimethylformamide (DMF) containing methylammonium chloride (MACl; 0.1 mol L^–1^) carried by continuous nitrogen flow over the film heated at 100 °C. The laminar flow of aerosol vaporizes near the film surface, and the vapor ingresses into the film. Figure 1a–c shows the scanning electron microscope (SEM) images of the MAPbI_3_ films before and after the treatment. The as-deposited MAPbI_3_ film comprises small grains (Figure 1a), while after 5 min of treatment, closely packed, monolithic grains are formed (Figure 1b). Overtreatment for 10 min results in the grains dewetting from the substrates, which leaves voids in the film (Figure 1c), strongly indicating the grain growth is driven by the minimization of surface energy.

In Figure 1d, we demonstrate the critical role of MACl as an additive in driving exceptional grain growth. An increase in the concentration of MACl in the DMF aerosol consistently increases the average lateral grain size of the MAPbI_3_ films (Figure S1) within the fixed time of 5 min. An analogous effect can be seen by incorporating similar AX compounds, where A = MA, formamidine (FA), or Cs and X = I or Br, Figures 1e and S2. Clearly, it is the A cation, instead of the halide, that drives exceptional grain growth, as potassium iodide (KI) shows no such effect since it cannot form a stable 3D ABX_3_ (here B = Pb) perovskite. MACl is employed in this study as we observed that its lower mass assists the formation of a consistent laminar aerosol flow, and it remains most stable in the DMF against continuous aerosolization.

The morphological change of MAPbI_3_ films and mechanism of grain growth are schematically illustrated in Figure 1f. The ingression of the DMF–MACl vapor is insufficient to fully dissolve the MAPbI_3_ films but enables the selective consumption of the smaller grains, allowing mass transfer of material from the smaller grains to the larger ones and eventually leading to an increase of overall grain size as time elapses, a process described by Ostwald ripening theory.^23^ Contrary to large perovskite grains grown from solution,^13,26,30^ the films remain in the solid state throughout our treatment (Figure S3). The absence of a liquid phase prevents additional perovskite nucleation on the surface; thus, the overriding process is the growth of the existing grains, leading to minimization of both surface energy and surface crystallographic defects.

While DMF vapor is known to break apart perovskite crystals,^29^ the role of MACl, as well as other AX (A = Cs, FA, MA, X = Br, I) additives, is to increase the solvating power of DMF, thereby driving continuous dissolution of the smaller grains among the existing perovskite grains. We think this is because the ingression of MA^+^, Cs^+^, or FA^+^ can distort the [PbI_6_]^4–^ octahedra in the MAPbI_3_ lattice and therefore facilitates the collapse of the 3D perovskite structures, analogous to the dissolution of MAPbI_3_ films by MA gas.^31^ It is worth noting that the additives are mostly expelled from the film when larger MAPbI_3_ grains are formed, as we observed a negligible band gap change that would be expected from incorporation of FA, Cs, Br, or Cl. (Figure S4).

**Figure 2:**
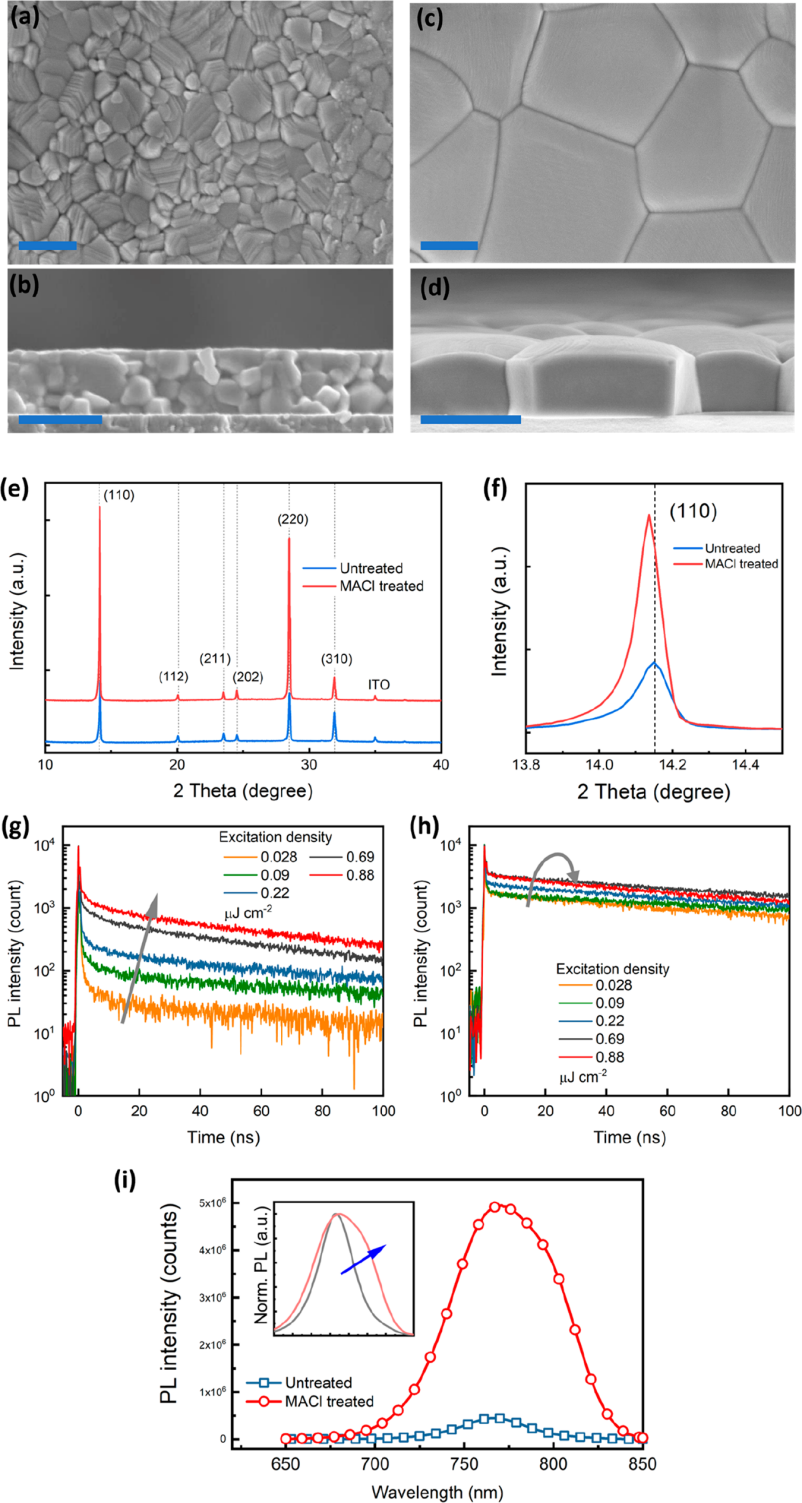
Characterization of MAPbI_3_ films. (a–d) Surface and cross-sectional SEM images of untreated MAPbI_3_ film (a, b) and MACl treated MAPbI_3_ film (c, d). Scale bars are 500 nm on all images. (e) X-ray diffraction patterns of untreated and MACl treated MAPbI_3_ films. (f) Zoom-in of the (110) diffraction peak. (g, h) Time-resolved PL spectra of untreated MAPbI_3_ (g) and MACl treated MAPbI_3_ (h) films, measured with a 435 nm pulsed laser under varied excitation density. (i) Steady-state PL spectra of MAPbI_3_ films measured with a 635 nm continuous-wavelength laser with an excitation density of 1.5 mW cm^–2^. The inset figure shows the spectra with normalized PL intensity, and the arrow indicates peak broadening of the MACl treated film.

The above results show that, by controlling the additives in the aerosol and the time of aerosol ingression, a remarkable grain size increase can be achieved while the film compactness is maintained. Hereon, we focus on the films before (denoted as “untreated”) and after 5 min of treatment with 0.1 M MACl in DMF (denoted as “MACl treated”). The surface and cross-sectional SEM images in Figure 2a–d further highlight the morphological difference. The large number of crystallographic terraces observed on the grains of the untreated film (Figure 2a), giving a “wrinkled” appearance, indicate the heterogeneous nature of the grain morphology due to rapid crystallization, as such a variety of crystallographic facets are exposed on the surface.^32^ The cross-sectional image further elucidates the heterogeneous morphology of the grains (Figure 2b). In contrast, the terraces are completely removed from the surface of the MACl treated film (Figure 2c). The cross-sectional image demonstrates the morphology of the monolithic grains that all have flattened side facets and smooth convex surfaces (Figure 2d), indicating comprehensive recrystallization at the surfaces and the grain boundaries.

X-ray diffraction (XRD) patterns, Figure 2e, show that the MACl treated film has a large increase of diffraction intensity, indicating improved crystallinity, and an approximate 4-fold increase of the relative intensity between the (110) and (310) peak, suggesting a strong preferential (110) crystallographic orientation. Closer inspection of the (110) XRD peak, Figure 2f, highlights a peak shift to lower 2θ angles after MACl treatment, indicating increased lattice spacing in the out-of-plane directions. This is typically ascribed to relaxation of the residual tensile strains in the in-plane direction,^33^ which is achieved through grain growth and morphological transition toward minimized surface energy.

To elucidate the change of charge recombination mechanisms, time-resolved photoluminescence (PL) spectroscopy was measured under varied excitation densities, Figure 2g–h. The PL decay dynamics of the untreated MAPbI_3_ film (Figure 2g) show typical biexponential behavior comprising a fast decay in the first few nanoseconds and a slow decay in tens of nanoseconds.^4^ Under the lowest excitation density, 0.028 μJ cm^–2^, the fast decay quenches over 99.5% of the initial PL, whereas increasing the excitation density consistently reduces the fast-decay component. Such excitation-dependent behavior is assigned to the filling of trap states by photogenerated carriers, and thus, the fast-decay component is ascribed to charge trapping.^34,35^ In contrast, the fast-decay quenches only around 80% of the initial PL in the MACl treated film under 0.028 μJ cm^–2^ (Figure 2h). As excitation density increases, the fast-decay magnitude shows much less change while the lifetime of slow decay becomes shorter, ascribed to the acceleration of band-to-band recombination as the density of photogenerated charge carriers increases. These results show the dominance of trap-assisted recombination in the untreated MAPbI_3_ film and that the density of trap states is substantially reduced by the MACl treatment.

Consistent with time-resolved PL data, the steady-state PL spectra show an approximate 12-fold increase of PL intensity by MACl treatment, Figure 2i. At the same time, the enhancement of PL emission is accompanied by a change of spectral shape, in that the peak becomes asymmetric and exhibits a “shoulder” on the low-energy side, shown in the inset figure. This indicates that the emissive film exhibits additional radiative recombination pathways with slightly reduced transition energy. We note that we observe a similar increase of grain size and spectral broadening of PL in a Cs_0.1_FA_0.9_Pb(I_0.95_Br_0.05_)_3_ film after MACl treatment (Figure S5), indicating MA is not a critical component for this effect to occur.

**Figure 3:**
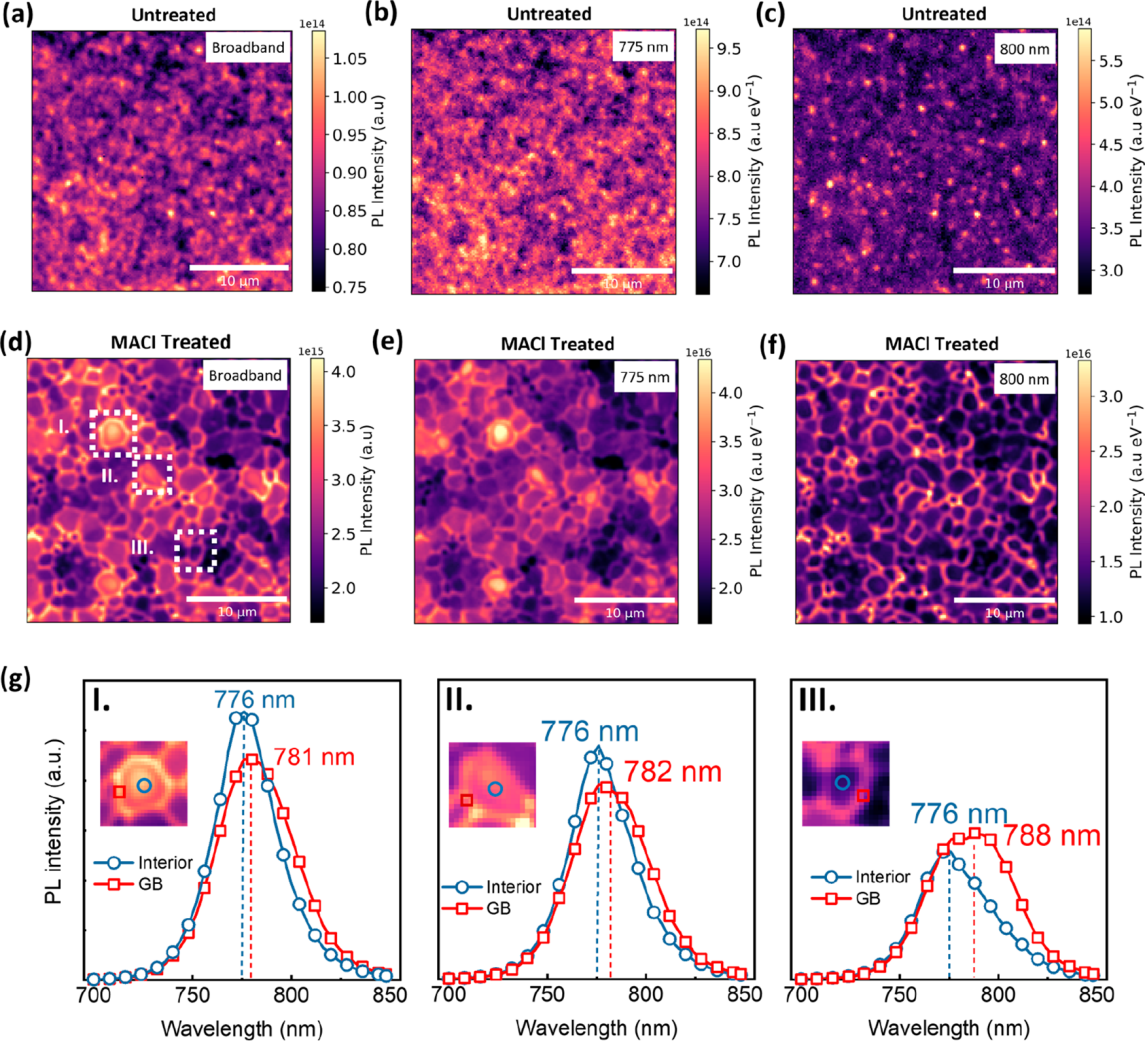
Photoluminescence mapping of MAPbI_3_ films. (a–f) Hyperspectral PL maps on an untreated MAPbI_3_ film (a–c) and a MACl treated MAPbI_3_ film (d–f) probed with broadband detector (a, d) and wavelength-selective detector at 775 nm (b, e) and at 800 nm (c, f). Maps were taken with 405 nm laser excitation with an intensity of 240 mW cm^–2^ in an ambient atmosphere. (g) Localized PL spectra from three different grains labeled “I”, “II”, and “III” in (d). On all images, a.u. refers to arbitrary units.

To understand the spatial origin of enhanced PL and the spectral “shoulder”, we turn to hyperspectral PL maps to probe local spectra. Figure 3 shows broadband emission maps and wavelength-specific emission maps at 775 and 800 nm from the untreated (a–c) and MACl treated films (d–f). The untreated film exhibits local variations of emission intensity in the broadband map (Figure 3a), but no clear trend in their spatial distribution is observed in wavelength-specific maps (Figure 3b,c), typical for perovskite films prepared with antisolvent washing.^36^ In the MACl treated film, we are readily able to resolve individual grains, as there is notably intensified PL along the GBs (Figure 3d). When one looks at the wavelength-specific maps, the brightening of the GB is less prominent in the 775 nm map (Figure 3e) but is the dominant origin of emission in the 800 nm map (Figure 3f), which coincides with the low-energy “shoulder” in the PL peak. We note that no such GB brightening is exhibited in films treated with DMF-only vapors (Figure S6), indicating the critical role that MACl plays in facilitating this behavior.

To gain further spectral insight, we plot in Figure 3g the local spectra at both the GB and grain interior from three representative grains (labeled “I” to “III” in Figure 3d), where the spectral difference between the GB and grain interior can immediately be seen. All these spectra show a redshift of peak position at the GB regions with the peak area comparable or even greater than the grain interiors, highlighting the emissive nature of GBs in MACl treated films. Therefore, we can conclude that the overall spectral asymmetry observed in Figure 2i associated with the emergence a low-energy subpeak stems directly from local brightening at the GBs in the MACl treated MAPbI_3_ film.

The observation of the red-shifted PL emission in the perovskite films has been ascribed to compression of the perovskite lattice and is usually accompanied by increased charge carrier lifetime,^33,37,38^ hereby signaling an improvement of film quality. The emergence of GB emission herein indicates a substantial defect elimination in these regions, while the redshift is likely correlated to a relaxation of tensile strains^39^ on the side facets of the monolithic grain. The latter is supported by the XRD data where relaxation of in-plane tensile strain leads to increased out-of-plane lattice spacing (Figure 2f). The grain edges show the largest improvement in optoelectronic properties as the GBs have maximum exposure to the DMF–MACl aerosol during the post-treatment. Hence, a complete recrystallization preferentially occurs at the edges of the existing grains, thereby eliminating the conventionally defective regions. We note that Cl passivation^40^ is not likely to play an overriding role in the formation of emissive GBs, as Cl-doping of MAPbI_3_ leads to an increase of the band gap whereas we observe a redshift. We also exclude the contribution of PL scattering at the grain edges to the spectral redshift/broadening by measuring probe-angle-dependent PL spectra, Figure S7, in which the change in spectral shape by varying the probing angle is negligible compared to the change induced by MACl treatment.

**Figure 4:**
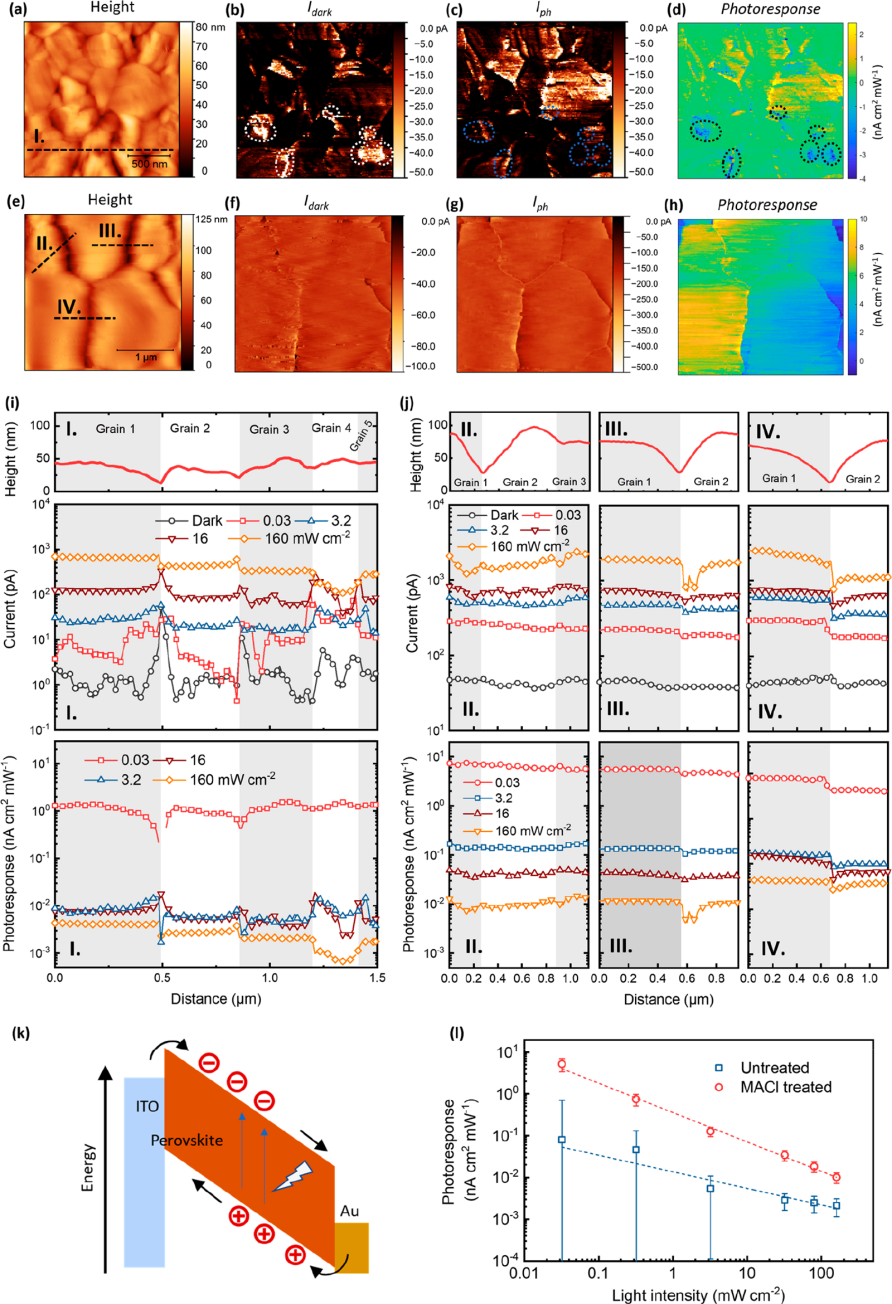
pc-AFM measurement of perovskite films. (a–h) pc-AFM maps of untreated MAPbI_3_ film (a–d) and MACl treated MAPbI_3_ film (e–h): surface height (a, e), dark current *I*_dark_ (b, f), and the photocurrent *I*_ph_ (c, g) and photoresponse (d, h) under 0.03 mW cm^–2^. Both films are deposited on ITO substrate. All *I*_dark_ and *I*_ph_ data are measured with an applied bias of −1.5 V on ITO. Illumination is provided by a 633 nm laser. (i, j) Line-scan data of height, *I*_dark_, *I*_ph_, and photoresponse taken from untreated film (marked “I”) and from three different positions in MACl treated film (marked “II”, “III”, and “IV”, respectively) under varied light intensities from 0.03 to 160 mW cm^–2^. (k) Schematic drawing of the energy diagram of the ITO/perovskite/Au heterojunction under bias. (l) Area averaged photoresponse as a function of light intensity. The error bars indicate spatial variation of the photoresponse within the measured area.

To further examine how the improved quality in the GB regions affects the local optoelectronic properties, we turn to photoconductive atomic force microscopy (pc-AFM) measurements. Figure 4a–h shows the maps for the untreated film (a–d) and the MACl treated film (e–h), including height (a, e), dark current (*I*_dark_) (b, f), photocurrent (*I*_ph_) (c, g), and photoresponse (d, h). Here, the photoresponse [nA cm^2^ mW^–1^] is defined as^41^
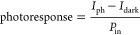
1and is used as an assay of photodetecting properties under certain incident light intensity (*P*_in_). We note that the direct estimation of the responsivity [A W^–1^] remains challenging due to the difficulty in determining the effective tip–sample emission area and thus the current density.^42^*I*_ph_ and photoresponse are mapped under varied *P*_in_ values between 0.03 and 160 mW cm^–2^ (Figures S8 and S9). Representative line-scan data taken from an untreated film (line “I”) and MACl treated film (lines “II”, “III”, and “IV”) are plotted in Figure 4i,j, respectively. Different grains along the line scans are indicated to highlight the intra- and intergrain variation of these quantities.

The map of *I*_dark_ highlights a substantial spatial inhomogeneity in untreated MAPbI_3_ (Figure 4b). The line-scan data (Figure 4i) further elucidate that the variation of *I*_dark_ is both intragrain and intergrain, but in general, *I*_dark_ tends to be greater near the GBs. When an illumination of 0.03 mW cm^–2^ is turned on, *I*_ph_ remains spatially inhomogeneous and does not fully track the distribution of *I*_dark_ (Figure 4c,i). We thus highlight a set of regions in Figure 4b–d that exhibit exceptionally high *I*_dark_ but do not show the proportionality of *I*_ph_ and thereby have a negligible photoresponse. Importantly, these regions showing a negligible photoresponse under these low-light levels serve as performance-limiting clusters in the untreated MAPbI_3_ in terms of photodetection. It can also be observed that these clusters tend to overlap certain crystallographic facets that are particularly defective. Note that these clusters all have a stronger photoresponse when the light intensity is increased, suggesting that the performance-limiting factor is trap-mediated charge recombination that prevails under weak illumination levels. This in itself is noteworthy, as it allows us to directly visualize trap-rich, performance-limiting regions of the perovskite films. In contrast, the MACl treated film exhibits remarkably improved spatial homogeneity of both *I*_dark_ and *I*_ph_, particularly under weak illumination levels as shown by the maps (Figure 4f,g) and line-scan data (Figure 4j). Importantly, the spatial variations of *I*_dark_ and *I*_ph_ are minimized both intergrain and intragrain in spite of the surface height change being greater than in the untreated film (Figure 4j). Therefore, the GBs in the MACl treated film become “invisible” in the electrical measurement under dark or weak illumination. The line-scan data also indicate that *I*_ph_ is much higher than *I*_dark_ as the illumination is applied, yielding stronger photoresponse in the MACl treated film.

We then turn to a quantitative analysis of the photoresponse of what is essentially an ITO/perovskite/Au photoconductor under bias, as indicated in Figure 4k. As Figure 4l shows, the photoresponse of both films decreases as light intensity increases, but the MACl treated films exhibit a substantially enhanced photoresponse; such an enhancement is much greater under lower light intensities, showing an increase by a factor of 20–40 in the range of 0.03–3.2 mW cm^–2^. We can also observe a much greater variation of both *I*_ph_ and photoresponse in the untreated film under lower light intensities, which tracks their large spatial inhomogeneity, indicating that the low *I*_ph_ and poor photoresponse are correlated to the large local variations of these quantities. These results highlight an exceptional improvement in low-light detecting capability using MACl treated films.

**Figure 5:**
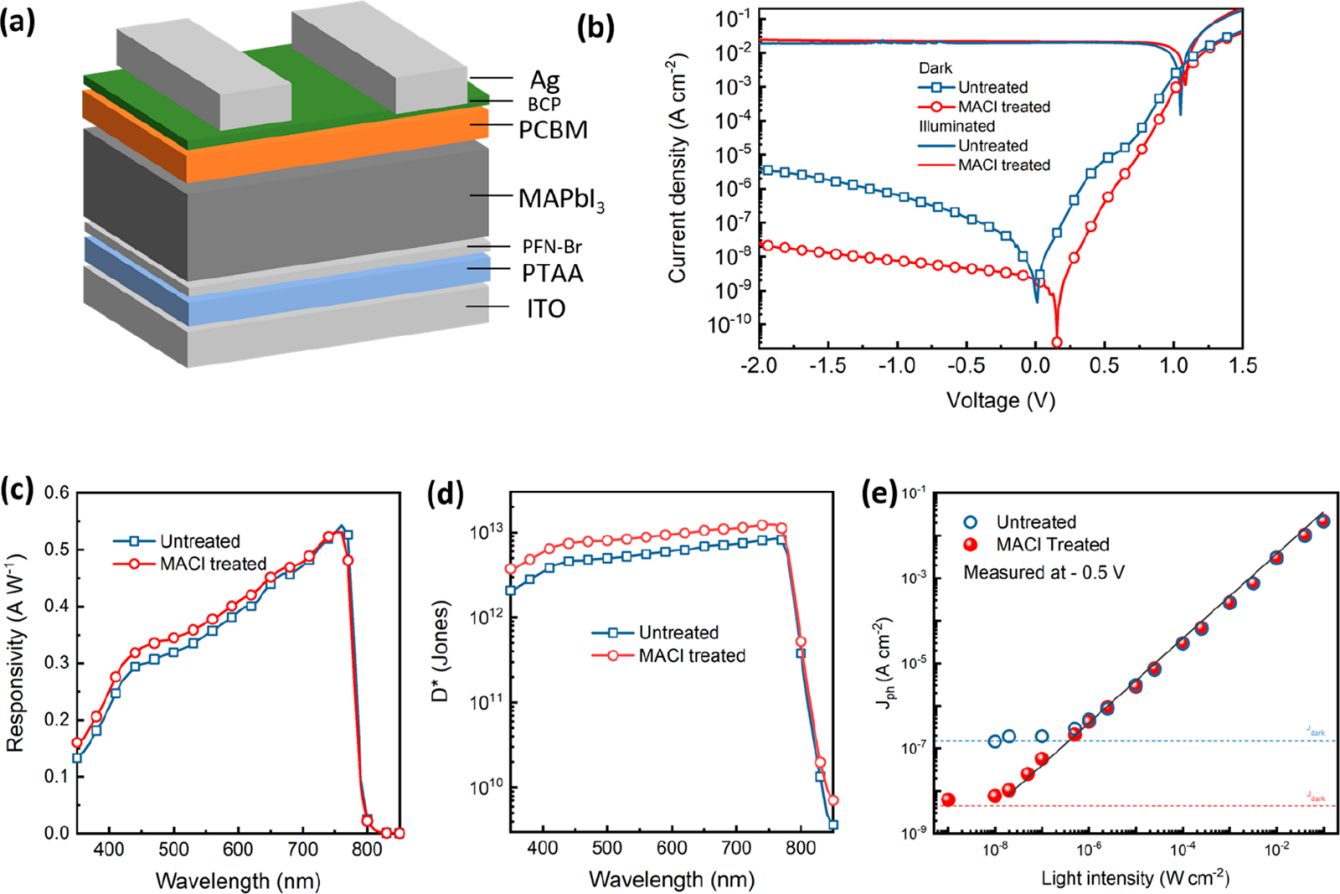
Characterization of the perovskite photodiodes (PPDs). (a) Schematic drawing of the architecture of the PPDs where poly[bis(4-phenyl)(2,4,6-trimethylphenyl)amine (PTAA) is used as the electron blocking layer, [6,6]-phenyl-C61-butyric acid methyl ester (PCBM) is used as the hole blocking layer, poly(9,9-bis(3′-(*N*,*N*-dimethyl)-*N*-ethylammoinium-propyl-2,7-fluorene)-*alt*-2,7-(9,9-dioctylfluorene))dibromide (PFN-Br) is used as the surface modifier of PTAA to reduce its hydrophobicity, and bathocuproine (BCP) is used as the interfacial dipole layer for the cathode. (b) Current density–voltage (*J*–*V*) scans of the PPDs under the dark and illumination of 100 mW cm^–2^ provided by an AM1.5G solar simulator. Scan rate is 25 mV s^–1^ in the forward direction from −2 to 1.5 V. (c) Responsivity spectra of the PPDs measured under an applied bias of −0.5 V. (d) Specific detectivity (D*) of the PPDs measured at −0.5 V, calculated from the responsivity and noise spectra. (e) Photocurrent measured at −0.5 V bias under varied light intensities provided by an AM1.5G solar simulator with neutral density filters. The solid line is a linear guideline, and the dashed lines indicate the *J*_dark_ of the respective PPDs.

Finally, to investigate how overcoming local inhomogeneities in MAPbI_3_ films translates to the performance of full optoelectronic devices, we fabricated and tested perovskite photodiodes (PPDs) with the architecture shown in Figure 5a. Figure 5b plots the current density–voltage (*J*–*V*) curves in the dark and under illumination (100 mW cm^–2^). As a key parameter governing the sensitivity of a photodiode, the dark current (*J*_d_) reported for PPDs varies in a wide range depending largely on the engineering of the contact layers and/or defects in the perovskite layer.^43^ We herein employ relative thick contact layers that are reported to suppress leakage current and thus reduce *J*_d_ (see Table S1 and Figure S10).^44^ Even for the PPD with an otherwise optimized layer thickness, MACl treatment brings about a remarkable reduction of *J*_d_. At −2 V, for example, *J*_d_ is 2.2 × 10^–8^ A cm^–2^ for the MACl treated PPD, about two orders lower than the *J*_d_ of 3.5 × 10^–6^ A cm^–2^ for the reference PPD. This is among the lowest *J*_d_ value reported for a PPD at this bias.^45^

Figure 5c plots the responsivity (*R*) of the PPDs calculated from the external quantum efficiency (EQE) as an assay of photon-to-electron conversion
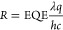
2where λ is the wavelength of incident light, *q* is the elementary charge, *h* is Planck’s constant, and *c* is the speed of light. We can observe a moderate increase of *R* in the MACl treated PPD, but the difference is less significant than that of *J*_d_. To properly calculate the specific detectivity (D*), we measured the noise power spectra for both PPDs, Figure S11. The noise floor is reached near 0.3 Hz, and the noise current (*i*_n_) at −0.5 V is approximately 1 order of magnitude lower in the MACl treated PPD, tracking the change of *J*_d_. Here, *i*_n_ comprises not only the shot noise from *J*_d_ but also the thermal noise from carrier agitation, but it is clear that the reduction of *i*_n_ can be attributed mostly to the reduction of *J*_d_. Using *R* and *i*_n_, we can determine D*, Figure 5d, which describes the sensitivity of PPD,
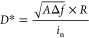
3where *A* is the pixel area and Δ*f* is electrical bandwidth. The MACl treated PPD shows higher D* over the whole spectrum with a peak value of 1.24 × 10^13^ Jones, while the peak value of D* for the reference device is 7.6 × 10^12^ Jones.

To highlight the improvement of low-light detection, *J*–*V* scans are performed under varied light intensities with *J*_ph_ at −0.5 V plotted in Figure 5e. The MACl treated PPD shows linearity between *J*_ph_ and light intensity down to approximately 2 × 10^–8^ W cm^–2^: 2 orders of magnitude lower than the value of 10^–6^ W cm^–2^ for the reference PPD. The improvement of low-light detection can be further confirmed by repeated *J*_ph_ measurements with increasing and decreasing light intensities (Figure S12). From these data we can calculate the linear dynamic range (LDR),
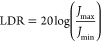
4where *J*_max_ (*J*_min_) is the maximum (minimum) value of the measured current. The LDR is 93 dB for the reference PPD and 126 dB for the MACl treated PPD. We can therefore conclude that the impact of our morphological modification on PPD is manifested by a remarkable reduction of *J*_d_ and thus *i*_n_, which enables a greater D* and extended LDR toward lower light levels. These improvements are consistent with the exceptional enhancement of the low-light photoresponse observed in the pc-AFM measurements on the bare perovskite films.

## Conclusions

In summary, we have demonstrated the inherent nanoscale inhomogeneity in antisolvent-assisted spin-coated perovskite films in which local defective clusters are significant and undermine the overall film quality. These performance-limiting clusters can be effectively eliminated using a vapor-mediated, post-annealing grain growth without the need of passivation agents. The facile treatment leads to remarkable photoluminescence observed at the grain boundaries, while the grain boundaries are almost invisible in the local photoconduction measurements, features which are atypical to polycrystalline thin films. The removal of local defective clusters is found to most significantly benefit the low-light photoresponse of perovskite films and perovskite photodiodes, enhancing their low-light detecting capabilities.

In a broader context, our findings highlight an issue that rapid crystallization of the perovskite film may have simultaneously created new challenges surrounding material uniformity and optoelectronic quality. The challenges not only undermine the low-light performance of these perovskites but also may limit their performance potential in photovoltaics (PV) or light-emitting diode (LED) devices. To this end, post-annealing grain growth can be an effective process to resolve this issue toward outstanding material quality and uniformity.

## Supporting Information Available

Experimental methods, SEM images, PL spectra, photocurrent and photoresponse maps, optimization of the layer thickness in the PPDs, noise power spectra of the PPDs, cyclic low-light *J*_ph_ measurements of the PPDs (PDF)
