# Supplementary material for: Overcoming Nanoscale Inhomogeneities in Thin-Film Perovskites via Exceptional Post-annealing Grain Growth for Enhanced Photodetection
Source: Nano Lett. 2022 Jan 21;22(3):979–88. doi: 10.1021/acs.nanolett.1c03839 (PMC9007526; doi:10.1021/acs.nanolett.1c03839)
Supplement: Supplementary file 1 — nl1c03839_si_001.pdf [file nl1c03839_si_001.pdf]

Overcoming Nanoscale Inhomogeneities in Thin-film Perovskites via Exceptional Post-Annealing Grain Growth for Enhanced Photodetection

Tian Du[1,5,,#], Filipe Richheimer[2], Kyle Frohna[3], Nicola Gasparini[4], Lokeshwari Mohan[1,5], Ganghong Min[4], Weidong Xu[4], Thomas J. Macdonal[1,4], Haozhen Yuan[1], Sinclair R. Ratnasingham[1,5], Saif Haque[4], Fernando A. Castro[2], James R. Durrant[4,6], Samuel D. Stranks[3,7], Sebastian Wood[2], Martyn A. McLachlan[5] and Joe Briscoe[1,*]*

[1]School of Engineering and Materials Science and Materials Research Institute, Queen Mary University of London, London E1 4NS, United Kingdom.
[2]National Physical Laboratory, Hampton Road, Teddington TW11 0LW, United Kingdom.
[3]Cavendish Laboratory, JJ Thomson Avenue, Cambridge CB3 0HE, United Kingdom.
[4]Department of Chemistry and Centre for Processable Electronics, Imperial College, London, W12 0BZ, United Kingdom.
[5]Department of Materials and Centre for Processable Electronics, Imperial College, London, W12 0BZ, United Kingdom.
[6]SPECIFIC IKC, College of Engineering, Swansea University, SA2 7AX, United Kingdom.
[7]Department of Chemical Engineering & Biotechnology, University of Cambridge, Philippa Fawcett Drive, Cambridge CB3 0AS, United Kingdom.

Corresponding authors:

[*]Email: j.briscoe@qmul.ac.uk
[*]Email: t.du@qmul.ac.uk.

Present address: [#]T.D.: Institute of Materials for Electronics and Energy Technology (i-MEET), Friedrich-Alexander University Erlangen-Nürnberg, Martensstraße 7, 91058 Erlangen, Germany. Email: tian.du@fau.de.

Supporting information

Experimental methods

Deposition of perovskite film

The precursor solution of $CH_3NH_3PbI_3$ ($MAPbI_3$) perovskite was prepared by co-dissolving equal molar ratio of lead iodide (PbI_2, 99.985%) and methylammonium iodide (MAI) in a mixed solvent of N,N-Dimethylmethanamide (DMF) and Dimethyl sulfoxide (DMSO) (9:1.1 in volume). The concentration of PbI_2 and MAI were both 1.5 M. The solution was stirred at 60°C until the precursors were fully dissolved. 40 μl precursor solution was dropped onto substrates with or without hole transport layers and was spun at 4000 rpm for 30s. At the seventh second, 0.5 ml diethyl ether was dripped onto the spinning substrate. The $MAPbI_3$ reference films were annealed on a hot plate at 100°C for 15 minutes. For MACl treatment the films were annealed for 5 minutes before transferring to a cylindrical chamber.

MACl treatment

The setup is modified from an aerosol assisted chemical vapor deposition system. Approximately 4 ml of MACl solution in DMF was aerosolized inside a bubbler by a piezoelectric ultrasonic generator. This was carried to a cylindrical chamber by nitrogen flow at 0.5 L min^{-1} through a planarizing baffle. The aerosol forms a laminar flow, steadily passes over the film and is drained on the other side of chamber. The substrate was heated at 100°C by a graphite block.

Structural and morphological characterization

X-ray diffraction (XRD) patterns of perovskite films were obtained with an X'pert Powder diffractometer (PANalytical), Cu Kα source. The diffraction patterns were measured over the range 7 - 40° 2θ, with the sample rotating during measurement. The top-view, tilt-angle and cross-sectional scanning electron microscopy (SEM) images were obtained using a LEO Gemini 1525 field emission gun scanning electron microscopy. The working voltage of SEM was fixed at 5 kV. To prevent charging, all

the films were coated with a 10 nm chromium layer.

Spectroscopic characterization

Steady-state photoluminescence (PL) spectra were measured with a commercial FL 1039 spectrometer (Horiba Scientific), measured with 635-nm monochromatic laser excitation at intensity of 1.5 mW cm^{-2}. PL decay was measured with a time-correlated single photon counting (TCSPC) spectrometer on a Delta Flex system (Horiba Scientific). The excitation was provided by a 435-nm monochromatic laser diode. The excitation density was measured by a power meter and was tuned by inserting neutral density optical filters.

Hyperspectral fluorescence mapping.

Hyperspectral photoluminescence maps were performed using an IMA^{TM} Vis wide-field optical microscope from Photon Etc. Samples were illuminated using a 405 nm continuous wave laser at an intensity of ~240 mW cm^{-2} through an Olympus 100× objective. A 415 nm long pass filter removed the laser emission from the collected light. The incident angle of the emitted photoluminescence onto a volume Bragg grating was varied to spectrally split the light incident onto the Hamamatsu CMOS camera. Reconstruction of the data allowed the extraction of photoluminescence spectra at each point on the sample.

Photoconductive AFM measurement

Photoconductive atomic force microscopy (pc-AFM) measurements were performed on an AIST-NT Combiscope 1000 AFM system. The AFM system was enclosed in a glovebox (Jacomex) under dry nitrogen conditions (~10 ppm O_2; 1 ppm H_2O). Au-coated 240AC-GG Opus probes from MicroMasch, with nominal resonance frequency at 70 kHz and 2 N/m force constant, were used for all experiments. Assuming nominal force constants, contact mode setpoint forces during pc-AFM were kept between 2 nN and 3 nN and no tip induced damage on the film surface was observed throughout measurements. The tip-at-the-front geometry of the selected probe model enabled

optical excitation without tip-shadowing.

Optical excitation was coupled in laterally using a 633 nm He-Ne laser beam. The lateral coupling angle was fixed at approximately 30° from the sample plane. The incident optical power was adjusted by placing a neutral density filter wheel in the beam path.

Fabrication and characterization of perovskite photodiode (PPD)

The PPDs were fabricated on indium-doped tin oxide (ITO) coated glass substrates. The substrates were sequentially cleaned in acetone, isopropanol, and deionized water (using ultrasonics) for 10 minutes followed by a N_2 dry. Prior to any deposition the substrates were treated by oxygen plasma for 10 minutes. Poly[bis(4-phenyl)(2,4,6-trimethylphenyl)amine (PTAA) (0.25 wt. % in chlorobenzene) was spin-coated onto the ITO at 5000 rpm or 3000 rpm for 20s. After drying for 1 minute, Poly [(9,9-bis(3'-(N,N-dimethylamino)propyl)-2,7-fluorene)-alt-2,7-(9,9–dioctylfluorene)] (PFN) (0.05 wt. % in methanol) was spin-coated onto the HTLs at 5000 rpm for 20s. All depositions were performed in ambient conditions prior to transfer to a N_2 filled glovebox. The perovskite films were deposited and treated as described above. Afterward, [6,6]-Phenyl-C61-butyric acid methyl ester (PCBM) (~ 80 nm in thickness) was deposited onto the perovskite layer by spin coating the solution (30 mg ml^{-1} in chlorobenzene) at 1200 rpm for 30s. Following that, bathocuproine (BCP) (0.5 mg ml^{-1} in methanol) was spin coated onto PCBM as interfacial dipole layer. Finally, 100 nm of Ag was thermally evaporated (1 Å/s) at a base pressure of 5×10^{-6} mbar.

Current density-voltage (J-V) characteristics of the PPDs were measured using a Keithley 2400 source meter, in the dark or illuminated by an AM 1.5 filtered xenon lamp (Oriel Instruments) at 1 sun intensity (100 mW cm^{-2}), calibrated using a silicon reference photodiode. The scan rate was fixed at 10 mV s^{-1} in both forward directions (-2 V to 2 V).

For determination of the Linear Dynamic Range (LDR), the light was attenuated using a selection of neutral density filters placed between the lamp and PPD. The photocurrent

(J_{ph}) was calculated as the difference in response between the illuminated current density (J_{light}) and dark current density (J_d) at each light intensity. Responsivity was measured using an integrated system from Quantum Design PV300. All the devices were tested in ambient air.

Supporting notes and figures

Role of halide additives in DMF

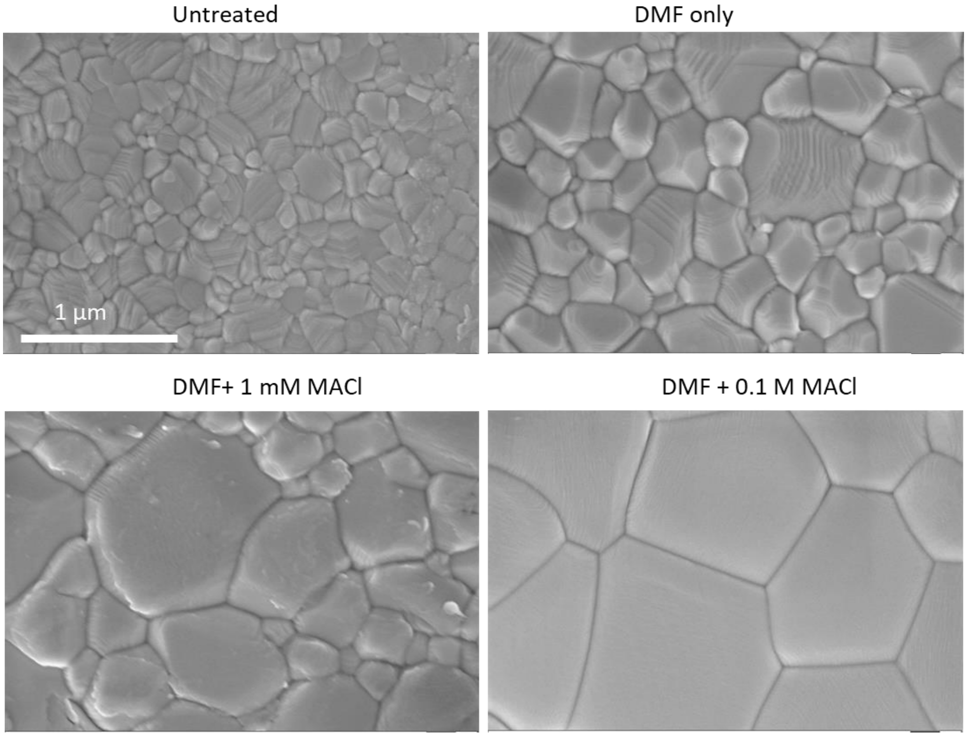

Figure S1. Surface SEM images of Perovskite films treated by DMF aerosol with different concentration of MACl.

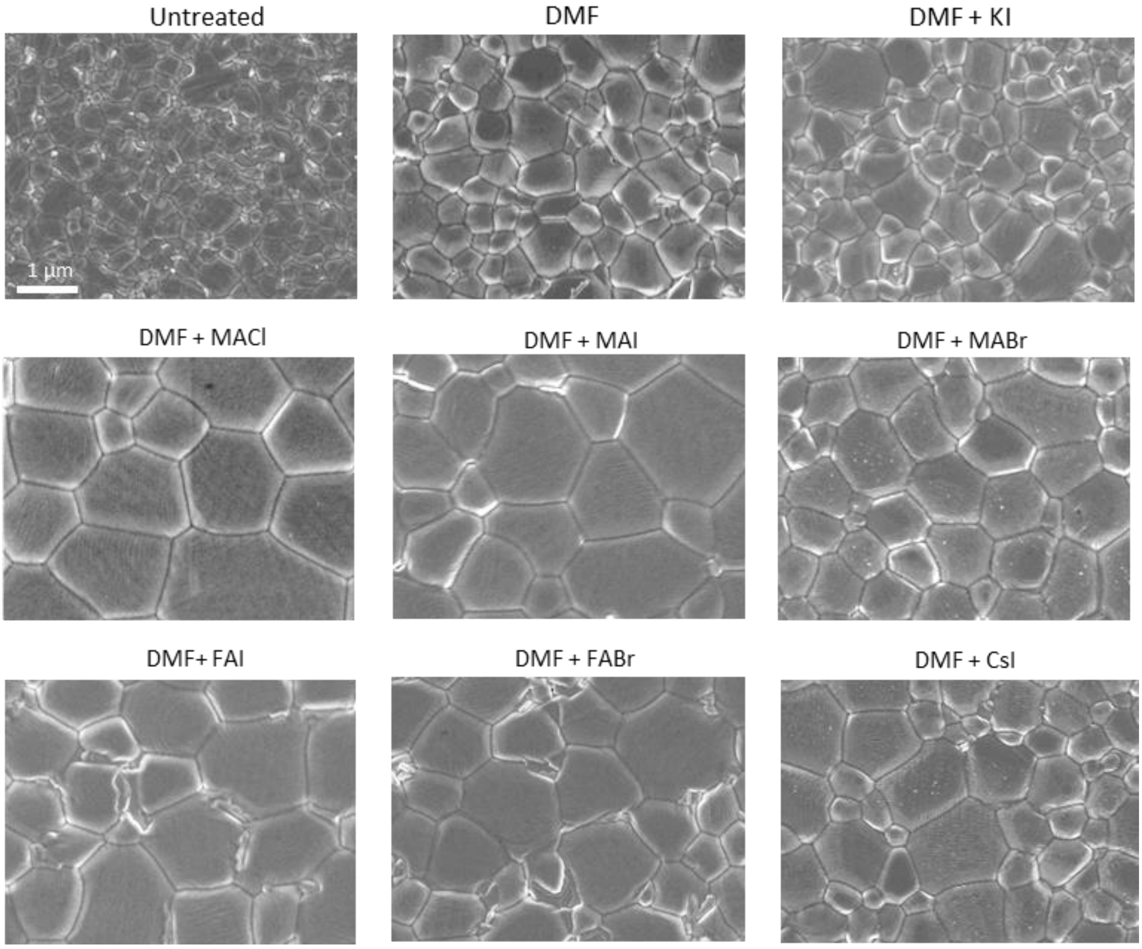

Figure S2. (a) Surface SEM images of perovskite films treated by DMF aerosol containing different halide salts, all at concentration of 0.1 M.

Solid-state crystal growth

Solid-state crystal growth can be achieved by maintaining the heating temperature of $MAPbI_3$ films at or above 100°C. Decreasing the heating temperature results in DMF completely "wetting" the film, and a discontinuous morphology is observed when the film is re-dried. These data show that it is only possible to obtain grain growth whilst maintaining compact film morphology when the film remains in the solid state.

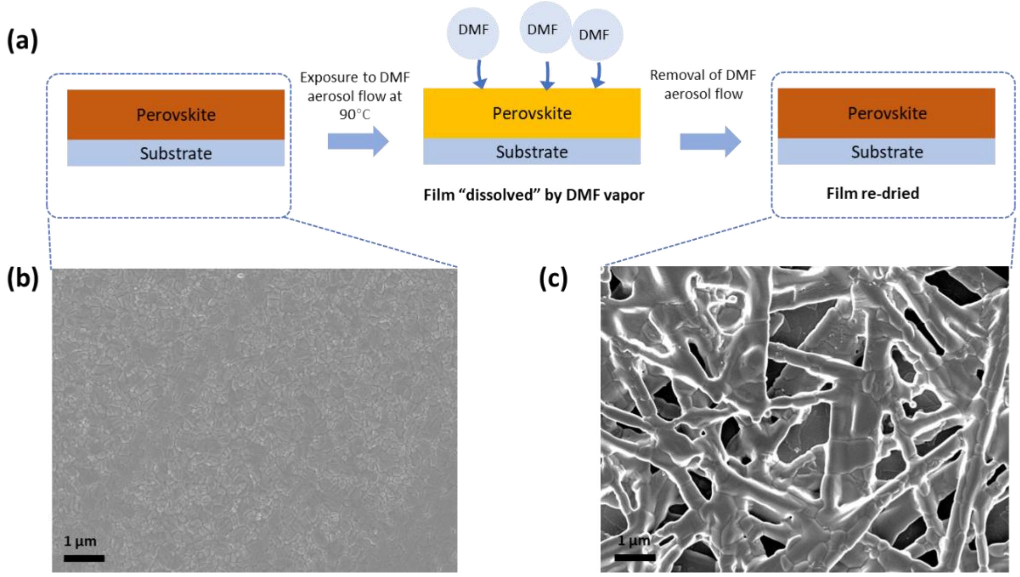

Figure S3. (a) Schematic drawing of partial dissolution of perovskite film by DMF vapor through reducing substrate heating temperature, and subsequent recrystallization of perovskite film when the aerosol is removed. **(b)** – **(c)** SEM images of reference perovskite film and a re-dried perovskite film after partial dissolution.

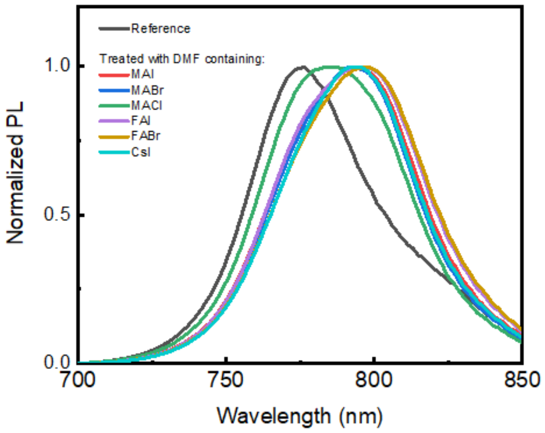

Figure S4. Steady-state PL spectra of perovskite films treated by DMF aerosol containing different halide salts.

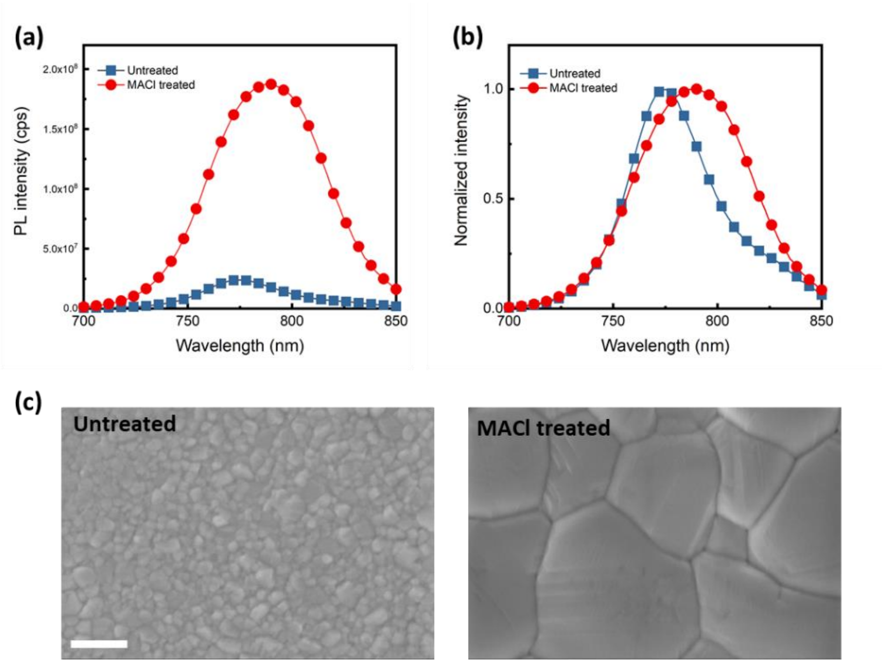

Figure S5. (a) Photoluminesce spectra of untreated and MACl treated $Cs_{0.1}FA_{0.9}Pb(I_{0.95}Br_{0.05})_3$ films. **(b)** Normalized PL spectra. **(c)** Surface SEM images of $Cs_{0.1}FA_{0.9}Pb(I_{0.95}Br_{0.05})_3$ films. Scale bar is 500 nm.

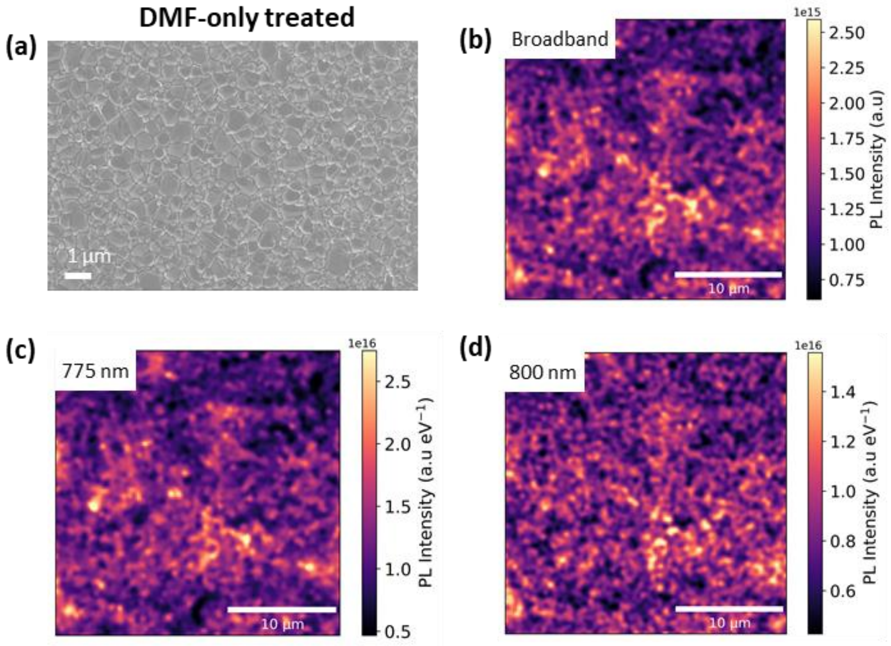

Figure S6. (a) Surface SEM images of a $MAPbI_3$ film treated by aerosolized DMF. **(b) – (d).** Hyperspectral PL maps of the DMF-only treated $MAPbI_3$ film probed with broadband detector (b) and wavelength-selective detector at 775 nm (c) and at 800 nm (d), respectively.

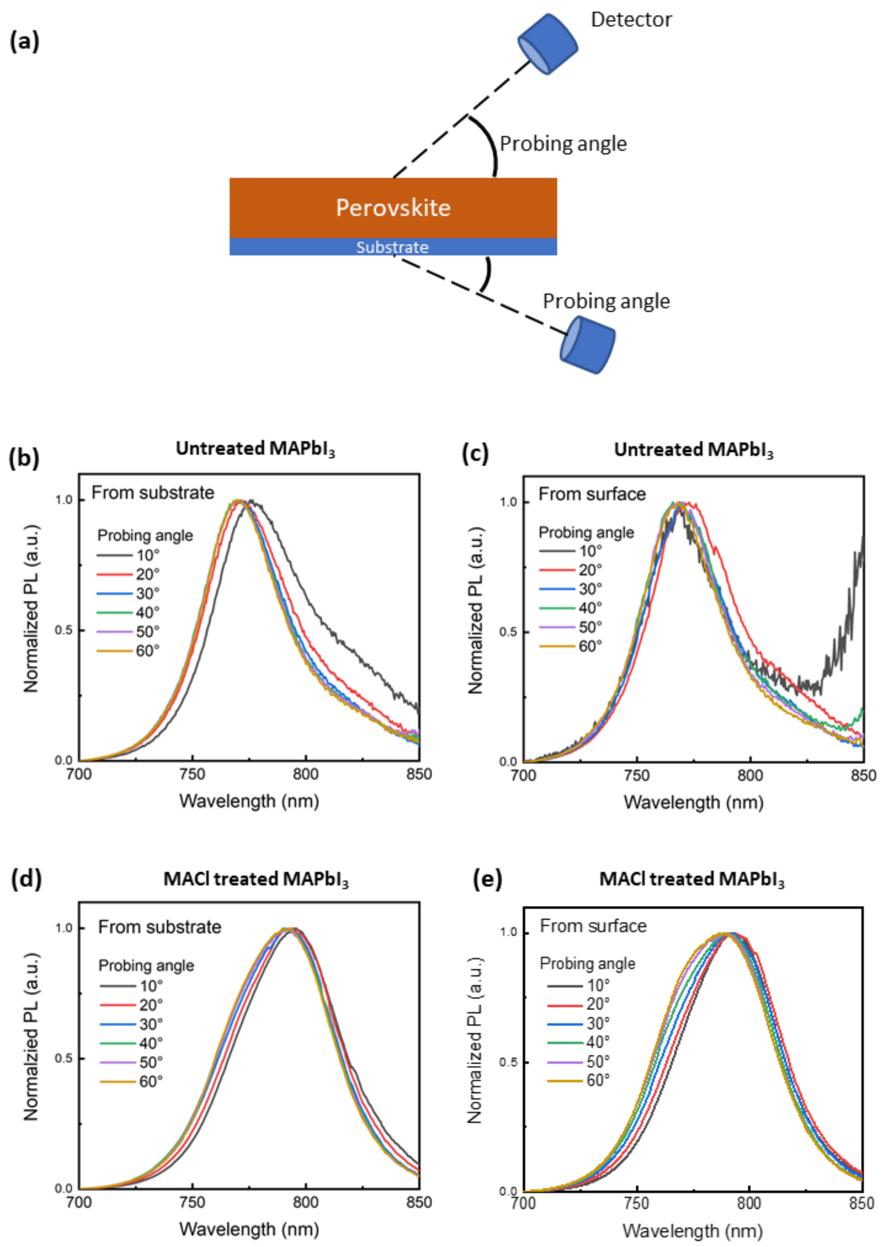

Figure S7. (a) Schematic drawing of PL set up with different positions of the detector. **(b)** PL spectra measured by varying the angle of the detectors with respect to the substrate, on both the surface side and the substrate side.

Pc-AFM measurement

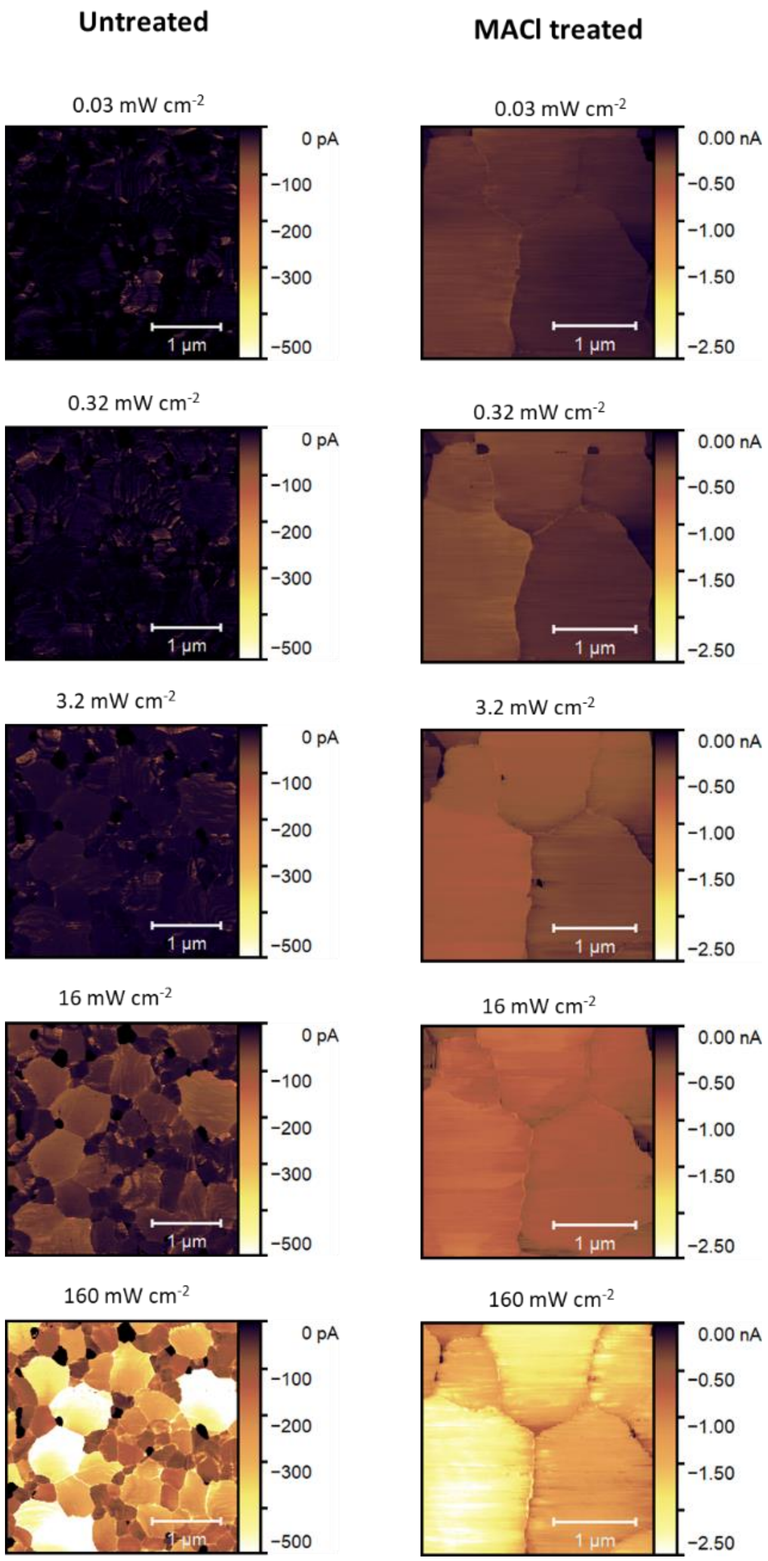

Figure S8. Photocurrent maps of reference perovskite and MACl treated perovskite film, measured under varied light intensities.

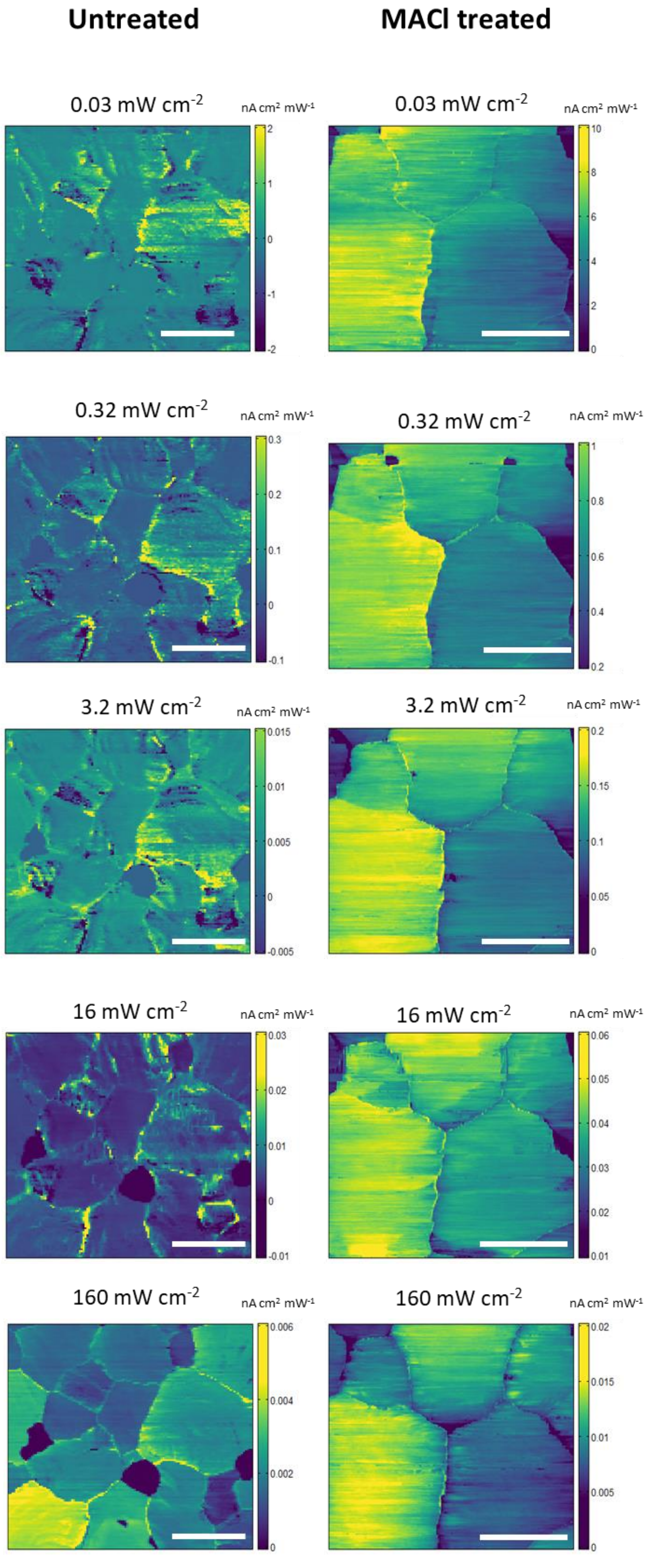

Figure S9. Photoresponse maps of reference perovskite and MACl treated perovskite film, measured under varied light intensities. Scale bars correspond to 1 μm.

Optimization of characterization of PPD

Table S1. Optimization of contact layer and perovskite layer of the PPD.

PPD type	HTL	Perovskite	ETL
A	PTAA, ~ 10 nm	200 nm	PCBM, ~40 nm
B	PEDOT:PSS	500 nm	PCBM, ~40 nm
C	PTAA, ~ 10 nm	500 nm	PCBM, ~40nm
D	PTAA, ~ 30 nm	500 nm	PCBM, ~ 80nm

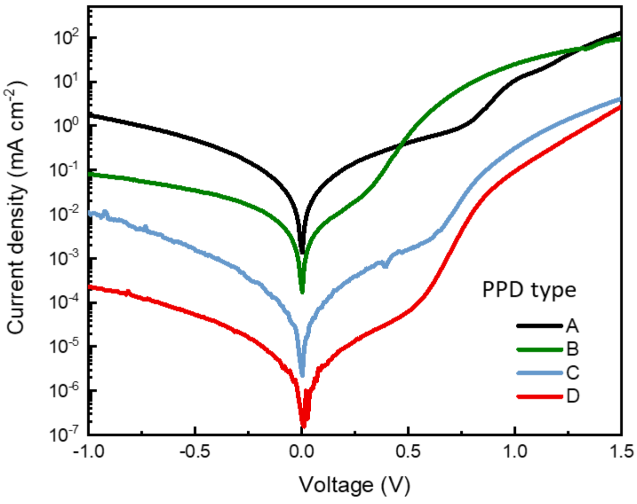

Figure S10. Dark current of different types of PPDs listed in Table S1.

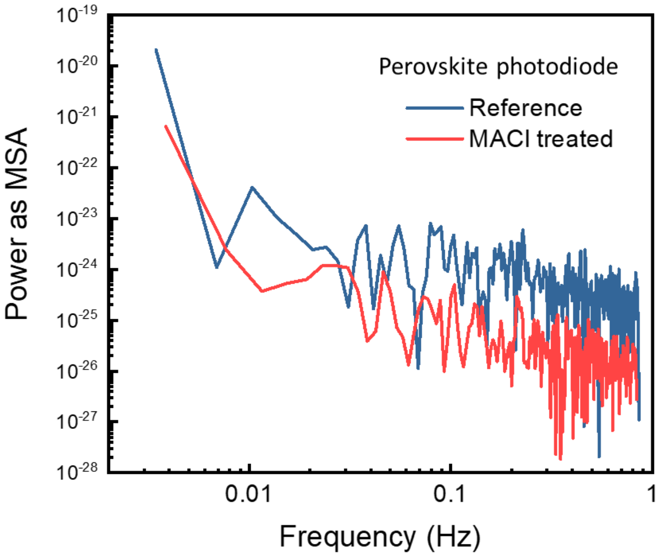

Figure S11. Noise power spectra of the PPDs.

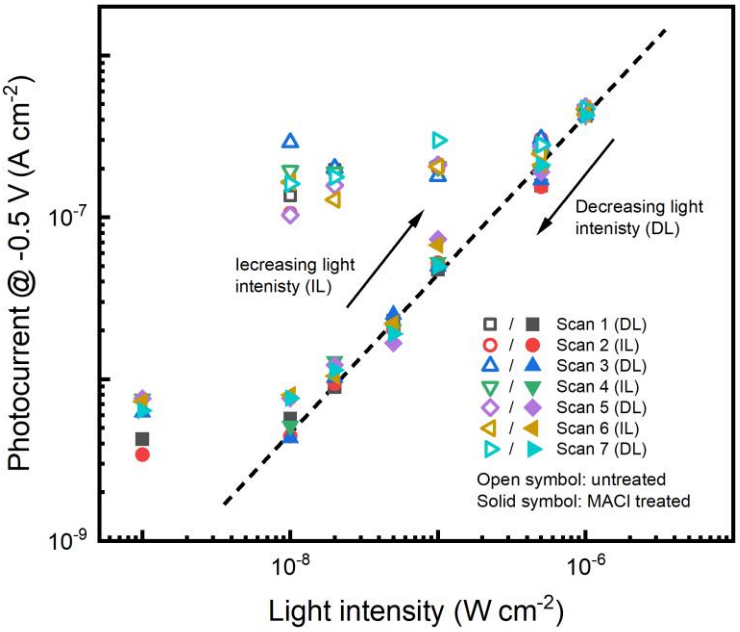

Figure S12. Repeated J_{ph} measurement (at – 0.5 V) with increasing and decreasing light intensities.
